# Independent and Mediated Associations of Adverse Childhood Experiences With Overweight and Obesity in Adults From the United States: Insights From the 2023 Behavioral Risk Factor Surveillance System (BRFSS) Data

**DOI:** 10.7759/cureus.93297

**Published:** 2025-09-26

**Authors:** Umer Maqsood, Shichen Zheng

**Affiliations:** 1 Research and Evaluation, California Rural Indian Health Board Inc., Roseville, USA

**Keywords:** adverse childhood experiences (aces), behavioral risk factors, chronic disease risk, obesity, socioeconomic factors

## Abstract

Adult overweight and obesity remain an urgent public health concern in the United States. While adverse childhood experiences (ACEs) are recognized as risk factors, it remains unclear whether their influence persists independently of adult socioeconomic and behavioral conditions. To our knowledge, this study is among the first to analyze the 2023 Behavioral Risk Factor Surveillance System (BRFSS) data with multilevel modeling to isolate ACE effects from adult risk factors. Using data from the 2023 BRFSS (n=269,521), this study employed multilevel logistic regression to examine the relationship between ACEs and adult overweight/obesity (BMI≥25), adjusting for demographic, socioeconomic, health access, behavioral, and chronic disease variables. ACEs were categorized as 0, 1-2, or ≥3.

Out of the total 269,521 respondents, 67.5% (n=182,008) were classified as overweight or obese. Participants with three or more ACEs (n=9,677) had a higher crude prevalence of overweight/obesity (69.7%) compared to those with no ACEs (n=12,786, 67.0%; p=0.02). However, in fully adjusted models, ACEs were not significantly associated with overweight/obesity (OR for ≥3 ACEs=1.08; 95% CI: 0.92-1.27). Independent predictors included race/ethnicity, income, education, physical inactivity, diabetes, and healthcare access. These findings suggest that ACEs may influence obesity indirectly, primarily through their impact on adult socioeconomic and behavioral factors. Interventions that address adult-level risk factors, particularly in populations with high ACE exposure, combined with trauma-informed care, may be more effective in reducing obesity prevalence.

## Introduction

Overweight and obesity are a concern for public health in the United States, as they impact a significant portion of American adults, over 70%. Specifically looking at obesity with a prevalence rate of over 42%, trends indicate a rise in both obesity and severe obesity from 1999 to 2018, with a brief slowdown observed between 2009 and 2012 [1-3]. These conditions contribute to a range of chronic diseases, including type 2 diabetes, cardiovascular disease, and certain cancers [4-6]. They are also associated with increased mortality and nearly $173 billion in annual healthcare costs [7].

The impact of overweight/obesity is not spread equally across the population, as it tends to affect those with lower education levels and income more significantly, along with certain racial and ethnic groups [8,9].

Additionally, there are differences in obesity rates based on geographic location; some Southern and Midwestern states show adult obesity rates over 35%, while Northeastern and Western states typically have lower rates [10]. These disparities highlight the importance of both individual-level and state-level factors in understanding the obesity epidemic.

These disparities underline the significance of considering both circumstances and state-level factors in addressing the issue of obesity in society. Literature often highlights the impact of early life challenges like adverse childhood experiences (ACEs) on adult weight issues like overweight or obesity. ACEs encompass adversities such as abuse (physical/sexual/emotional), neglect by parents or caregivers, parental separation, household substance abuse, and exposure to violence [11]. Such experiences are associated with disruptions in metabolic balance, immune system functioning, and health-related habits [12,13]. Meta-analyses have shown that individuals exposed to four or more ACEs have 1.4 to 1.6 times the odds of obesity in adulthood compared to those without such exposures [14, 15]. Notably, these associations appear stronger among women, particularly in relation to childhood sexual or physical abuse [16].

Proposed mechanisms associated with ACEs to adult obesity include chronic activation of the hypothalamic-pituitary-adrenal (HPA) axis, increased inflammatory load, and maladaptive coping behaviors such as emotional eating, reduced physical activity, or substance use [12,17]. Moreover, ACEs are associated with comorbid depression and anxiety, which independently contribute to obesity risk [18].

Despite this compelling evidence, most studies to date examine ACEs in isolation or focus on psychological outcomes [12,17,18], rather than integrating early adversity with adult-level social and behavioral predictors in models of obesity. Thus, it remains unclear whether ACEs contribute to unique risk above and beyond current adult circumstances.

Adult-level predictors, such as education, income, housing, and health behaviors, are well-established correlates of overweight/obesity. Lower socioeconomic status is consistently associated with higher obesity prevalence, particularly among women [9,19]. Physical inactivity and poor diet are direct contributors to weight gain, and their prevalence is disproportionately high in lower-income communities [20]. Barriers to healthcare access, including lack of insurance or a regular provider, have also been shown to limit opportunities for weight management and preventive care [21].

However, few investigations have examined both early-life disadvantage and contemporaneous adult-age risk factors at the same time, especially with multilevel, population-representative data. Furthermore, much of the literature focuses solely on obesity (BMI ≥30), overlooking the substantial population classified as overweight (BMI 25-29.9), who also face elevated health risks and are at high risk of progressing to obesity [1,22]. Including both overweight and obesity provides a more comprehensive picture of weight-related health risks and can inform earlier interventions.

This study will address these gaps by leveraging nationally representative data from the Behavioral Risk Factor Surveillance System (BRFSS), which includes data on ACEs, sociodemographic and behavioral risk factors, and self-reported height and weight across all 50 U.S. states. Using a multilevel framework (individuals nested within states), this study will explore how ACEs and adult-level risk factors jointly predict overweight/obesity and whether ACEs retain predictive value after accounting for adult conditions. In the BRFSS, ACEs are assessed through self-reported exposure to eight adversities before age 18, including physical, emotional, or sexual abuse; neglect; parental separation or divorce; household mental illness; substance abuse; incarceration; and exposure to domestic violence. Each affirmative response is scored, producing a cumulative ACE score ranging from zero to eight.

Using nationally representative 2023 BRFSS data, this study aims to determine whether ACEs are independently associated with overweight/obesity (BMI ≥25) among U.S. adults, after accounting for sociodemographic, socioeconomic, behavioral, and health-related factors.

We hypothesize that higher ACE exposure (≥3 ACEs) will be associated with greater odds of overweight/obesity compared to no ACE exposure, even after adjustment for adult-level covariates [15,23,24].

## Materials and methods

The 2023 BRFSS is administered by the Centers for Disease Control and Prevention (CDC). It is an annual cross-sectional survey that collects data on health-related risk behaviors and health conditions from non-institutionalized adults 18 and older within 50 states, the District of Columbia, and U.S. territories. The response rate varies by state and territory. In 2023, the overall response rate was 44.7%, ranging from 21.7% to 63.1%, according to the CDC [25]. Responses for all variables marked as “Don’t know” or “Refused” were treated as missing data and handled by listwise deletion.

Each reported adverse ACE was assigned one point, resulting in a total possible score ranging from zero to eight. Based on the number of ACEs reported, scores were grouped into three categories: none, one to two, and three or more. Individuals with three or more ACEs were classified as having a high ACE score, while those with one or two were considered to have a low score. This method of categorization aligns with the Life Course Metrics Project’s recommended indicator for ACEs among adults and has been applied in previous state-level research [24]. Overweight/obesity was the outcome of interest. It was based on the calculated variable from the survey “Adults who have a body mass index greater than 25.00” and was categorized into yes and no.

Covariates include demographic variables, chronic health conditions, health care access, and physical activity. Demographic variables comprised state of residence, income level, age, sex, race/ethnicity, and educational attainment. States were grouped into geographic regions according to the U.S. Census Bureau: Northeast, Midwest, South, and West. Income was categorized based on prior literature and grouped into three categories: low income (less than $25,000), middle income ($35,000 to $74,999), and high income ($75,000 or more). Age was grouped into six categories: 18-24, 25-34, 35-44, 45-54, 55-64, and 65 years or older. Sex was categorized as male or female. Race/ethnicity was classified as White, Black, Hispanic, Native Hawaiian, Asian, or Other individuals (including Pacific Islander, multiracial, and individuals identifying as “something else”). Educational attainment was defined as either less than high school (did not graduate from high school) or more than high school (graduated from high school, attended or completed college or technical school).

Chronic health conditions included diabetes mellitus status, coronary heart disease (CHD) or myocardial infarction (MI), and chronic obstructive pulmonary disease (COPD) or chronic bronchitis. Diabetes mellitus status was based on self-reported responses to the question “Have you ever been told you have diabetes?” and categorized as: yes; yes, but only during pregnancy (for females); no (including prediabetes or borderline diabetes); or don’t know/not sure. CHD or MI status was based on self-reported history of either condition and categorized as either reporting CHD/MI or not reporting either. COPD or chronic bronchitis status was determined by responses to the question “Have you ever been told you have COPD, emphysema, or chronic bronchitis?” and categorized as yes or no.

Health care access was assessed through three indicators: health insurance coverage, having a personal health care provider, and the ability to afford medical care. Health insurance coverage was determined by whether the respondent reported having any form of health insurance and was categorized as yes or no. Having a personal health care provider was assessed with the question “Do you have one person (or a group of doctors) that you think of as your personal health care provider?” and categorized as: yes, only one provider; yes, more than one provider; or no. The ability to afford medical care was based on whether, in the past 12 months, the respondent needed to see a doctor but could not due to cost, and was categorized as yes or no.

The percentage of students who are overweight/obese by ACE status, as well as demographic variables, chronic health conditions, health care access, and physical activity, was calculated. Group differences were first evaluated using Rao-Scott chi-square tests. Bivariate associations between ACEs, covariates, and overweight/obesity were first evaluated using Rao-Scott chi-square tests. All covariates were retained for inclusion in regression models based on prior literature and p-value thresholds. Multiple logistic regression models were used to examine the association between ACE status and overweight/obesity status, adjusted for demographic variables, chronic health conditions, health care access, and physical activity. Collinearity was assessed by examining correlation coefficients and variance inflation factors (VIFs) among key predictors; no values exceeded accepted thresholds. Although the conceptual framework suggests ACEs influence socioeconomic and behavioral factors that in turn affect obesity, we still conducted collinearity checks to ensure stable estimates in multivariable models. Model 1 adjusted for the above-mentioned variables, including race/ethnicity, state, education level, having own or renting a home, income level, age, gender, exercise in the past 30 days, diabetes mellitus status, CHD/MI status, COPD or chronic bronchitis status, whether they have a personal health care provider, and length of time since last routine checkup.

Model 2 was adjusted for ACE status additionally. We used SAS version 9.4 (SAS Institute, Cary, NC) for all analyses. All analyses incorporated BRFSS survey weights, strata, and primary sampling units (PSUs).

## Results

Among the total sample of 269,521 individuals, 67.53% (n=182,008) were classified as overweight or obese. Individuals with three or more ACEs (n=9,677; 69.69%) had a higher prevalence compared to those with no adverse experiences (n=12,786; 66.96%) (p=0.02).

Prevalence of overweight or obesity also varied significantly across demographic and health-related characteristics, including region, education, home ownership, age, sex, physical activity, chronic conditions, race/ethnicity, and others (Table 1)

**Table 1:** Percentage of overweight or obese by demographic characteristics (BRFSS 2023, n=269,521) Weighted prevalence of overweight/obesity (BMI ≥25) by demographic, social, and behavioral characteristics among U.S. adults (BRFSS 2023, n=269,521). Percentages are weighted estimates of population prevalence. P-values are from Rao–Scott chi-square tests comparing prevalence across subgroups. A significant p-value indicates differences in overweight/obesity prevalence across categories of the given variable. * indicates a statistically significant value. CHD: coronary heart disease; MI: myocardial infarction; COPD: chronic obstructive pulmonary disease; BRFSS: Behavioral Risk Factor Surveillance System; ACE: adverse childhood experience

Factors	Overweight/obesity (%)	P-value
Total	269521 (67.53%)	
Adverse Childhood Experience		
0 ACEs	12786 (66.96%)	0.020*
1–2 ACEs	13583 (68.75%)	
3+ ACEs	9677 (69.69%)	
Demographic Factors		
State		
Northeast	77269 (69.94%)	< 0.0001
Midwest	46400 (64.48%)	
Other	5516 (70.99%)	
South	71945 (68.97%)	
West	68391 (64.64%)	
Education Level		
High school or less	83094 (68.54%)	< 0.0001
More than high school	185735 (66.94%)	
Income Level		
Low income	32270 (67.63%)	< 0.0001
Middle income	95821 (70.05%)	
High income	101609 (68.35%)	
Age		
Age 18 to 24	11300 (46.03%)	< 0.0001
Age 25 to 34	26294 (64%)	
Age 35 to 44	35713 (71.98%)	
Age 45 to 54	40254 (76.33%)	
Age 55 to 64	52233 (75.39%)	
Age 65 or older	101171 (67.75%)	
Gender		
Male	140742 (71.76%)	< 0.0001
Female	128779 (63.24%)	
Race		
White	74432 (68.45%)	< 0.0001
Black	9123 (76.45%)	
Hispanic	4983 (76.19%)	
Asian	914 (45.67%)	
Native Hawaiian	995 (76.05%)	
Other	1464 (70.69%)	
Social Factors		
Own or Rent Home		
Own	192703 (69.79%)	< 0.0001
Rent	63876 (65.23%)	
Other arrangement	11504 (53.91%)	
Don't know/not sure	487 (54.65%)	
Have Any Health Insurance		
Yes	247701 (68.17%)	0.087
No	12930 (66.82%)	
Have a Personal Health Care Provider		
Yes, only one	148574 (68.67%)	< 0.0001
More than one	89711 (68.83%)	
No	29127 (61.71%)	
Length of Time Since Last Routine Checkup		
Within past year	221069 (69.12%)	< 0.0001
Within past 2 years	22169 (62.63%)	
Within past 5 years	12046 (62.32%)	
5 or more years ago	10191 (62.66%)	
Don't know/not sure	2484 (58.3%)	
Never	1340 (62.92%)	
Could Not Afford To See a Doctor		
Yes	22879 (67.5%)	0.914
No	245889 (67.56%)	
Behavioral Factors		
Exercise in Past 30 Days		
Yes	197985 (65.82%)	< 0.0001
No	70867 (72.99%)	
Diabetes Mellitus		
Yes	46238 (83.93%)	< 0.0001
Yes, but female told only during pregnancy	1960 (68.57%)	
No	212874 (64.7%)	
No, pre-diabetes or borderline diabetes	7983 (82.21%)	
Don't know/not sure	406 (66.75%)	
Ever Had CHD or MI		
Reported having MI or CHD	25127 (75.01%)	< 0.0001
Did not report having MI or CHD	241745 (67%)	
Length of Time Since Last Routine Checkup		
Yes	21495 (71%)	< 0.0001
No	246910 (67.3%)	
Don't know /not sure	1067 (66.97%)	

Table 2 presents the initial model (Model 1) and the adjusted model (Model 2) predicting overweight/obesity. In Model 1, demographic factors such as age and race/ethnicity, as well as socioeconomic factors (income, education), were strong predictors of overweight/obesity. Behavioral (physical inactivity) and chronic disease variables (diabetes mellitus, CHD/MI, COPD) were also significant. In Model 2, after adding ACEs, the associations for demographic and behavioral factors persisted with little attenuation. Individuals with one to two ACEs (OR=1.09; p=0.265) and those with three or more ACEs (OR=1.08; p=0.361) did not exhibit a statistically significant increase in odds of being overweight or obese compared to those with no ACE exposure. Black individuals (OR=1.44; p=0.0002) remained significantly associated with higher odds of overweight/obesity, while Asian individuals (OR=0.52; p=0.001) and females (OR=0.81; p=0.002) remained significantly associated with lower odds.

**Table 2:** Multivariable logistic regression predicting odds of overweight or obesity (BRFSS 2023, n=269,521) Multivariable logistic regression models estimating adjusted odds ratios (ORs) and 95% confidence intervals for overweight/obesity (BMI ≥25) among U.S. adults (BRFSS 2023, n=269,521). Model 1 includes demographic, socioeconomic, behavioral, health condition, and healthcare access variables. Model 2 adds ACEs to assess their independent association after adjustment. * indicates a statistically significant value. CHD: coronary heart disease; MI: myocardial infarction; COPD: chronic obstructive pulmonary disease; BRFSS: Behavioral Risk Factor Surveillance System; ACE: adverse childhood experience

Factors	Model 1		Model 2	
Adverse Childhood Experience				
ACEs (ref=No)				
1–2 ACEs			1.09 (0.94, 1.26)	0.265
3+ ACEs			1.08 (0.92, 1.27)	0.361
Demographic Factors				
Race/Ethnicity (ref=White)				
Asian	0.39 (0.32, 0.47)	< 0.0001*	0.52 (0.35, 0.78)	0.001*
Black	1.44 (1.3, 1.6)	< 0.0001*	1.44 (1.19, 1.74)	0.0002*
Hispanic	1.63 (1.42, 1.86)	< 0.0001*	1.32 (0.98, 1.79)	0.071*
Native Hawaiian	1.43 (1.07, 1.9)	0.015*	1.13 (0.46, 2.74)	0.792
Other	1.14 (0.91, 1.42)	0.2583	1.25 (0.83, 1.89)	0.295
State (ref=Northeast)				
Midwest	1.16 (1.1, 1.23)	< 0.0001*	NA	
South	1.11 (1.04, 1.18)	0.002*	1.16 (1.02, 1.32)	0.026*
West	0.96 (0.87, 1.05)	0.3304	1.12 (0.9, 1.4)	0.294
Education Level (ref=High school or less)				
More than high school	0.91 (0.85, 0.98)	0.008*	0.82 (0.7, 0.95)	0.009*
Age (ref=18 to 24)				
Age 25 to 34	2.08 (1.82, 2.39)	< 0.0001*	2.09 (1.55, 2.83)	< 0.0001*
Age 35 to 44	2.76 (2.4, 3.16)	< 0.0001*	2.82 (2.07, 3.85)	< 0.0001*
Age 45 to 54	3.25 (2.8, 3.78)	< 0.0001*	2.77 (2, 3.82)	< 0.0001*
Age 55 to 64	3.06 (2.64, 3.54)	< 0.0001*	2.57 (1.87, 3.54)	< 0.0001*
Age 65 or older	1.72 (1.5, 1.98)	< 0.0001*	1.74 (1.29, 2.35)	0.0003*
Gender (ref=Male)				
Female	0.65 (0.61, 0.7)	< 0.0001*	0.81 (0.71, 0.93)	0.002*
Social Factors				
Own or Rent Home (ref=Own)				
Rent	0.96 (0.88, 1.04)	0.305	1.02 (0.85, 1.22)	0.866
Other arrangement	0.8 (0.68, 0.93)	0.004*	0.66 (0.47, 0.93)	0.019*
Don't know/not sure	1.72 (0.92, 3.23)	0.090	3.37 (0.74, 15.28)	0.116
Income Level (ref=Low income)				
Middle income	1.24 (1.13, 1.37)	< 0.0001*	1.22 (0.99, 1.5)	0.061
High income	1.19 (1.07, 1.32)	0.002*	1.22 (0.99, 1.52)	0.068
Have a Personal Health Care Provider (ref=No)				
Yes, only one	1.11 (0.99, 1.24)	0.065	1 (0.79, 1.27)	0.988
More than one	1.1 (0.98, 1.23)	0.120	1.06 (0.83, 1.36)	0.648
Length of Time Since Last Routine Checkup (Ref=Never)				
Within past year (anytime less than 12 months ago)	1.8 (1.25, 2.6)	0.002*	1.89 (0.72, 5)	0.198
Within past 2 years (1 year but less than 2 years ago)	1.59 (1.1, 2.31)	0.015*	2.01 (0.75, 5.36)	0.163
Within past 5 years (2 years but less than 5 years ago)	1.51 (1.03, 2.22)	0.034*	1.54 (0.57, 4.17)	0.400
5 or more years ago	1.44 (0.99, 2.11)	0.059	1.51 (0.55, 4.12)	0.423
Don't know/not sure	0.99 (0.59, 1.67)	0.973	0.93 (0.28, 3.1)	0.910
Behavioral Factors				
Exercise in Past 30 Days (ref=No)				
Yes	0.71 (0.65, 0.77)	< 0.0001*	0.77 (0.65, 0.91)	0.002*
Diabetes (ref=No)				
Yes	2.53 (2.25, 2.83)	< 0.0001*	2.37 (1.94, 2.9)	< 0.0001*
Yes, but female told only during pregnancy	1.53 (1.12, 2.09)	0.008*	1.45 (0.72, 2.92)	0.2947
No, pre-diabetes or borderline diabetes	2.41 (1.86, 3.12)	< 0.0001*	2.26 (1.43, 3.57)	0.001*
Don't know/not sure	1.37 (0.62, 3.02)	0.437	1.89 (0.54, 6.6)	0.3192
Ever Had CHD or MI (ref=No)				
Reported having MI or CHD	0.98 (0.85, 1.13)	0.820	1.02 (0.8, 1.3)	0.8802
Ever Had COPD, Emphysema, or Chronic Bronchitis (Ref=No)				
Yes	0.92 (0.82, 1.04)	0.175	0.95 (0.76, 1.19)	0.6423
Don't know/not sure	0.48 (0.24, 0.96)	0.037*	0.07 (0.01, 0.41)	0.003*

## Discussion

In this study, we analyzed data from the 2023 BRFSS survey and found that 67.53% of the 269,521 individuals sampled were classified as overweight or obese. ACEs were significantly associated with a higher likelihood of being overweight or obese. However, this association was no longer significant after adjusting for current sociodemographic and behavioral factors, suggesting that the effects of ACEs are not independent of these variables.

Our result is similar to the previous literature examining ACEs and obesity using 2011 BRFSS data, which supported the association that high ACEs are associated with increased odds of obesity [26]. However, this association is no longer significant after adjusting for demographic and socioeconomic factors. This aligns with our finding that ACEs did not independently predict obesity once current conditions were considered.

The attenuation of the ACE-obesity association after adjustment indicates that adult conditions and behaviors are critical intervention points. This suggests that even for individuals with high ACE exposure, improving their current socioeconomic and lifestyle factors could substantially reduce their risk of obesity. In practical terms, policies that expand access to resources like nutritious food, safe places to exercise, quality education, and stable employment opportunities in communities with high ACE prevalence might mitigate the long-term health impacts of early trauma [18]. By addressing these social determinants (many of which were significant in our analysis), we can indirectly buffer the pathway from ACEs to obesity. The CDC has emphasized that creating safe, stable, nurturing relationships and environments for children, essentially preventing ACEs from occurring, could potentially avert a portion of the burden of adult chronic conditions, including overweight/obesity [27]. Our results support this upstream approach: prevention of childhood adversity is a worthy goal not only for its own sake but also because it may reduce downstream health issues. However, our study also highlights that current adversity (like adult poverty or lack of healthcare) is strongly tied to obesity. This underscores the need for integrated strategies that combine ACE prevention with tackling adult risk factors. Thus, while we work to reduce childhood trauma exposure for future generations, we must concurrently invest in helping those already affected by trauma to lead healthier lives.

There are some demographic and socioeconomic factors associated with obesity. Obesity prevalence was higher among non-Hispanic Black and Hispanic individuals, consistent with national disparities associated with socioeconomic, cultural, and environmental influences [28]. According to CDC data from 2017-2020, obesity prevalence was highest among Black adults (49.9%), followed by Hispanic adults (45.6%), with White adults at 41.4% [28]. Age was another key factor, with middle-aged adults showing the highest obesity rates, while younger and older adults had lower prevalence, mirroring national data and suggesting life stage-specific interventions [28]. Gender differences were subtler overall, though women were more likely to fall into the severe obesity category, reflecting both biological and behavioral dynamics. Lower income and educational level proved influential predictors, with scarce access to healthful foods, physical activity opportunities, and healthcare increasing risk for obesity, particularly among those experiencing socioeconomic distress. Lack of physical activity was strongly associated with obesity, and trauma-exposed individuals (particularly women) may face additional barriers to regular exercise. Diabetes also showed a strong association with obesity, underlining the clinical importance of weight management. Notably, lack of healthcare access, such as being uninsured or lacking a regular provider, was associated with higher obesity prevalence, likely reflecting broader social disadvantage and missed opportunities for prevention and treatment. These results underscore the importance of comprehensive, equity-oriented public health approaches that consider both present conditions and the ACEs in addressing obesity.

One clear policy implication is the potential value of trauma-informed public health interventions. Trauma-informed care, originally developed in behavioral health, is an approach that acknowledges the pervasive impact of ACEs on individuals’ well-being and behavior. Applying this lens to obesity prevention could mean designing programs that are sensitive to the needs and triggers of people with trauma histories. For example, weight-loss or fitness programs could incorporate psychological support or stress-management training, recognizing that emotional regulation difficulties stemming from ACEs might lead to overeating or low motivation for exercise. Healthcare providers could be trained to screen for ACEs during routine visits (including obesity-related counseling sessions) in order to tailor advice appropriately. Our findings suggest that ACEs alone did not predict obesity after accounting for other factors, which might suggest that routine ACE screening in obesity clinics would identify many patients whose weight issues are entangled with factors like depression, smoking, or socioeconomic stress, all of which require comprehensive treatment plans. Indeed, researchers have advocated for improved screening and detection of trauma in healthcare settings as a means to improve downstream health outcomes related to eating behavior and weight. By knowing a patient’s trauma history, a clinician might, for instance, recommend trauma-focused therapy or ensure the patient has access to social services while simultaneously addressing weight management. The potential benefit is twofold: addressing trauma-related mental health needs can improve overall self-care, and doing so in tandem with obesity treatment might yield better adherence and outcomes. There is precedent for this approach: pilot interventions have shown that incorporating trauma-informed principles (e.g., building trust, patient empowerment, avoiding traumatization) in programs for chronic disease management can improve patient engagement.

One major strength is the use of the 2023 BRFSS data, which is a large, nationally representative sample of U.S. adults. The BRFSS is the world’s largest continuously conducted health survey system, completing over 400,000 interviews per year across all 50 states. Utilizing this dataset allowed our analysis to have adequate statistical power and broad generalizability to the adult population. The inclusion of ACE-related questions in the 2023 survey provided a unique opportunity to examine trauma history in a diverse community sample, rather than in clinical or high-risk samples used in some earlier ACE studies.

Limitations

Our study has limitations. First, the cross-sectional design of BRFSS means we cannot infer causality or temporal order between ACEs and obesity (or between obesity and the covariates). While it is reasonable to assume ACEs occurred in childhood and preceded adult obesity, the timing of obesity onset is not captured; some individuals may have already been obese in adolescence, potentially before or concurrently with certain ACE exposures. We also cannot determine whether the adult risk factors (e.g., low income, smoking, depression) arose as consequences of ACEs or whether they independently influenced obesity. Longitudinal data would be needed to unravel these temporal sequences and mediation pathways. Second, reliance on self-reported data introduces several concerns. All variables were self-reported by respondents via telephone. Self-report is subject to recall bias and social desirability bias. Third, unmeasured and residual confounding is a concern. We adjusted for many variables, but there are other factors we could not include due to data limitations. For example, we did not have direct measures of diet (caloric intake or diet quality), an important determinant of obesity. It’s plausible that ACEs influence dietary habits (e.g., greater consumption of comfort foods or sugary drinks as coping mechanisms), and if we had dietary data, we might find that accounting for it further explains the ACE-obesity association. Residual confounding (e.g., diet, mental health) remains possible. ACEs were categorized cumulatively; subtype-specific effects may be obscured.

**Figure 1:**
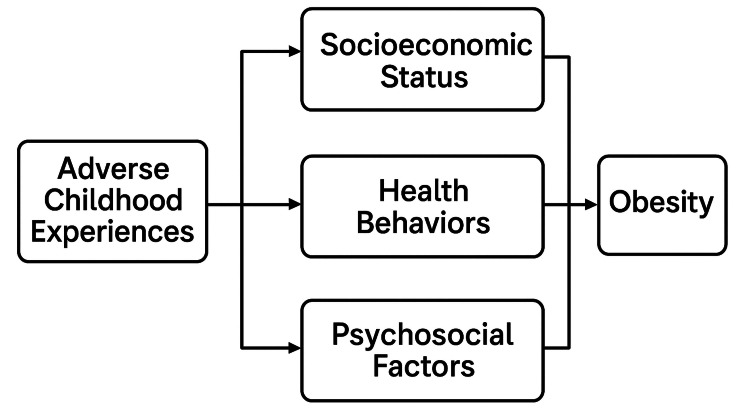
Conceptual framework Conceptual framework illustrating hypothesized direct and indirect pathways linking ACEs to adult overweight or obesity. (Source: Authors’ original work)

The conceptual framework (Figure 1) illustrates the hypothesized relationships between ACEs and adult overweight/obesity within a life-course epidemiological context. ACEs are positioned as early-life exposures that may influence adult obesity directly through biological embedding and chronic stress pathways and indirectly via adult socioeconomic status and behavioral risk factors.

In this model, adult socioeconomic conditions and health-related behaviors are included as both mediators and potential confounders, reflecting their dual role. Individuals with higher ACE exposure may be more likely to experience socioeconomic disadvantage or engage in adverse health behaviors in adulthood, both of which are independently associated with higher obesity risk.

By structuring our analysis to sequentially adjust for these adult factors, we aim to assess whether ACEs have an independent effect on adult obesity or whether their influence is explained through downstream social and behavioral pathways. The framework supports this approach by mapping the directionality and complexity of these relationships, reinforcing the need for a multidimensional analysis strategy that spans early-life and adult determinants of health.

This study opens several avenues for future investigation. To build on our findings, longitudinal research will be especially important. A key next step is to conduct prospective cohort studies that follow individuals from childhood (when ACEs occur) into adulthood to observe the development of obesity over time. Another important direction is researching the specific types of ACEs and their differential impacts. Our analysis treated ACEs cumulatively, but not all ACEs are equivalent in how they might lead to obesity. Future studies should examine which subtypes of adversity are most obesogenic.

## Conclusions

In conclusion, our analysis of the 2023 BRFSS data found that the association between ACEs and adult obesity was no longer significant after adjusting for current sociodemographic and behavioral factors. This aligns with prior research, suggesting ACEs primarily influence obesity through adult socioeconomic and lifestyle factors. Policy implications emphasize improving current socioeconomic conditions and adopting trauma-informed approaches in obesity prevention and healthcare. Future longitudinal research should clarify causal pathways and explore specific ACE subtypes to inform targeted interventions.
